# Is a Heavy–Slow Resistance Exercise Program an Appropriate Treatment Approach for All Patients with Lateral Elbow Tendinopathy? Editorial

**DOI:** 10.3390/jcm11061556

**Published:** 2022-03-11

**Authors:** Dimitrios Stasinopoulos

**Affiliations:** Member of Laboratory of Neuromuscular & Cardiovascular Study of Motion (LANECASM), Deptartment of Physiotherapy, Faculty of Health and Caring Sciences, University of West Attica, Agiou Spyridonos 28, Egaleo, 12243 Athens, Greece; dstasinopoulos@uniwa.gr

Heavy–slow resistance exercise programs are the most effective physiotherapy treatment approaches in lateral elbow tendinopathy (LET) management. However, many physiotherapists recommend neuromobilization, myofascial trigger point therapy, and cervical/thoracic mobilization, adjunct to a heavy–slow resistance exercise program in LET treatment. The questions that arise are when and why physiotherapists recommend the above three recommended physiotherapy maneuvers in LET, a painful overuse tendon condition.

The above three physiotherapy techniques are recommended in persistent LET (PLET). However, the use of the term PLET is not clear in the literature, since the medical society does not provide a definition for the term PLET. The use of the term PLET in the literature ranges from several (4–5) weeks to many (5–6) months after the first onset [1].

Physiotherapists recommend the above three physiotherapy methods in PLET because it is necessary to avoid marking PLET solely as a tendinopathy. PLET is a disorder with the involvement of the biochemical milieu at the extensor digitalis communis and the radial nerve [2]. In addition, myofascial trigger points co-exist with PLET, causing it or predisposing patients to it [3]. Finally, patients with PLET present cervical and thoracic spondylosis, even though the role of cervical and thoracic spine spondylosis in the prognosis of PLEP requires validation [4,5].

According to previous reported issues, a heavy–slow resistance exercise program cannot be used as monotherapy for the treatment of LET when LET is persistent. Neuromobilization, myofascial trigger point therapy, and cervical/thoracic mobilization have to be used as adjuncts to a heavy–slow resistance exercise program for the management of PLET.

The aim of this editorial is to encourage questions about the optimal treatment approach for PLET management, and help clinicians. A debate on this topic is most welcome.
